# ‘Opening up the mind’: problem-solving therapy delivered by female lay health workers to improve access to evidence-based care for depression and other common mental disorders through the Friendship Bench Project in Zimbabwe

**DOI:** 10.1186/s13033-016-0071-9

**Published:** 2016-05-11

**Authors:** Melanie Abas, Tarryn Bowers, Ethel Manda, Sara Cooper, Debra Machando, Ruth Verhey, Neha Lamech, Ricardo Araya, Dixon Chibanda

**Affiliations:** Psychology and Neuroscience, Institute of Psychiatry, King’s College London, London, UK; Zimbabwe AIDS Prevention Project, Department of Community Medicine, University of Zimbabwe, Harare, Zimbabwe; University of Cape Town, Cape Town, South Africa; Women’s University in Africa, Harare, Zimbabwe; Schizophrenia Research Foundation (SCARF), Chennai, India; Centre for Global Mental Health, London School of Hygiene and Tropical Medicine, London, UK; Zimbabwe Ministry of Health, Harare Central Hospital, ST 14 Southern, Harare, Zimbabwe

**Keywords:** Evidence-based intervention, Depression, Common mental disorders, Problem-solving therapy, Friendship bench, Zimbabwe, Low income countries

## Abstract

**Background:**

There are few accounts of evidence-based interventions for depression and other common mental disorders (CMDs) in primary care in low-income countries. The Friendship Bench Project is a collaborative care mental health intervention in primary care in Harare for CMDs which began as a pilot in 2006.

**Case presentation:**

We employed a mixture of quantitative and qualitative approaches to investigate the project’s acceptability and implementation, 4–8 years after the initial pilot study. We carried out basic descriptive analyses of routine data on attendance collected between 2010 and 2014. We also conducted five focus group discussions (FGDs) with LHWs in 2013 and 12 in-depth interviews, six with staff and six with patients, to explore experiences of the intervention, which we analysed using grounded theory. Results show that the intervention appears highly acceptable as evidenced by a consistent number of visits between 2010 and 2014 (mean 505 per year, SD 132); by the finding that the same team of female community LHWs employed as government health promoters continue to deliver assessment and problem-solving therapy, and the perceived positive benefits expressed by those interviewed. Clients described feeling ‘relieved and relaxed’ after therapy, and having their ‘mind opened’, and LHWs describing satisfaction from being agents of change. Characteristics of the LHWs (status in the community, maturity, trustworthiness), and of the intervention (use of locally validated symptom screen, perceived relevance of problem-solving therapy) and continuity of the LHW team appeared crucial. Challenges to implementation included the LHWs ongoing need for weekly supervision despite years of experience; the supervisors need for supervision for herself; training needs in managing suicidal and hostile clients; poor documentation; lack of follow-up of depressed clients; and poor access to antidepressants.

**Conclusions:**

This case study shows that a collaborative care intervention for CMDs is positively received by patients, rewarding for LHWs to deliver, and can be sustained over time at low cost. Next steps include evaluation of the impact of the intervention through a randomised trial, and testing of a technological platform for supporting supervision and monitoring clients’ attendance.

## Background

There have been repeated calls to improve access to evidence-based interventions for depression and other common mental disorders (CMDs) through primary care in low-income countries [1]. However, there are very few published accounts of such interventions. Of the few randomised controlled trials within Africa of therapies for CMDs, most have been conducted either in specialised populations (e.g. maternal depression, HIV care settings) [2] and/or in partnership with NGOs outside government public health systems [3] and it is unknown if they have been translated into delivery by existing staff in general primary care settings. Task-shifting is a way to increase coverage, but it is unknown if lay health workers (LHWs) working routinely in government systems in low-income countries can deliver evidence-based therapies for CMDs.

The aim of this study is to report on a primary care mental health project called the ‘Friendship Bench’. This comprises a structured collaborative care intervention for CMDs, including screening and evidence-based psychological therapy provided by trained and supervised lay community health workers attached to primary care. Stepped care and referral options are included in the package. The ‘Friendship Bench’ itself is a specially made wooden bench placed on the grounds of the three primary care health (PHC) clinics where the screening and psychological therapy takes place. The name ‘Friendship Bench’ derives from the Zimbabwean Shona term *Chigaro Chekupanamazano* that translates as ‘bench to sit on to exchange ideas’. The project, which uses materials from previous cross-cultural research in the country [4, 5], was started by a local psychiatrist (DC) in 2006 in response to a request from stakeholders in the local district community for a ‘no-cost’ community mental health intervention [6].

## Case presentation

The aims of this case-study are to learn lessons about the acceptability and implementation of a collaborative care intervention driven by trained and supervised existing community health lay workers in a low-income setting in Sub-Saharan Africa. This would inform potential scale-up through existing platforms, both in Zimbabwe and for other low-income settings, and also inform ways to expand the intervention into new sites ahead of a randomised clinical trial in Zimbabwe [7].

### Original intervention, 2006–2008

The delivery of the original intervention and preliminary evidence of its efficacy have been described in detail elsewhere [6]. The LHWs cadre, known as ‘*ambuya utano’* (grandmother health providers) are employed by the City of Harare Health Education Unit to work on the ground delivering health promotion to prevent disease outbreaks and promote health. They form part of a network of 400 health promoters city-wide, all female, who are organised geographically and trained and supervised by 15 district health promotion officers. Within the Zimbabwean context, health promotion is viewed as a ‘caring’ profession and recruitment was initially conducted on a voluntary basis. In line with public perception of this work, applicants to these posts have traditionally been female.

Referrals to the Friendship Bench come from primary care, community agencies such as the police, and self-referral. Primary care nurses who suspect a patient has significant emotional distress advise them to sit on the wooden bench in the clinic grounds to discuss their problems with the LHW on duty. The LHWs themselves also attract referrals by giving talks on stress, depression and ‘thinking too much’ [8]—a local idiom for emotional distress—to the crowds sitting in queues for routine primary health care. In summary, the LHW sits with the patient on the wooden bench and carries out a structured psychosocial assessment, including socio-demographic information, the presenting problems [4], mental health symptoms using the Shona Symptoms Questionnaire (SSQ) for CMDs [5] and a brief risk screen. Following this, the LHW can give advice and discharge, begin problem-solving therapy (PST), invite back for up for further sessions of PST for those scoring above the SSQ cut-point, and/or refer to the supervisor if the SSQ is very high or in the presence of other risk. For clients in dire financial need, the LHW can also refer to a small number of income generating projects, including one started by the Friendship Project called Zee Bag [9]. Zee Bag comprises group support for depressed females with school age children. It is facilitated by ex-patients, an artist and a psychologist and includes teaching craft skills, such as crocheting of bags from recycled plastic that could be used to make items to sell.

### Project changes between 2009 and 2012

Between 2009 and 2012 changes from the original pilot [6] included loss of the study nurse and psychologist who originally supervised the LHWs, and reduction of the psychiatrist visits from weekly to monthly. In 2010, a female volunteer counsellor took on supervision of the LHWs. Partnership with a local University enabled internships for trainee psychologists. These interns helped out with data management and with supporting the counselling supervisor. In the 2006 version of the intervention the LHWs were trained to provide 6 sessions of PST and then refer those who remain above cut-point on the SSQ to their supervisor and/or direct to the psychiatrist [10]. Partnerships with a Diaspora health charity (Zimbabwe Health Training Support, ZHTS) and with the Institute of Psychiatry, London, enabled booster training in PST and simple behavioural activation techniques. This led to further structuring of the existing intervention, for example providing written prompts to draw out client-generated solutions and to encourage the client to re-start behaviours that they had found positive in the past. Funds for the programme fluctuated depending on donations. When funds were available the supervisor received travel expenses and/or salary (up to US$500 per month), the intern received a small stipend for data management, and the LHWs received US $6–10 per month in recognition of conducting mental health work on top of their basic work. We set out to gather the data for this case study in 2013 to help inform a planned randomized controlled trial of the intervention [7].

## Methods

To investigate acceptability and implementation we aimed to analyse routine data on attendance and on aspects of the stepped care system between 2008 and 2014. To further investigate acceptability we gathered data from LHWs and clients through five Focus Group Discussions (FGDs) with LHWs (8 to 12 per group) and in-depth interviews with staff (5 with LHWs and 1 with their supervisor) and six patients in 2013. The draft interview/FGD guides were informed by pilot work and through gaining access to pre-published versions of systematic literature reviews [11, 12]. As well as asking about experiences of and challenges in delivering/receiving the intervention, questions also covered understandings of CMDs, and, for staff, perceptions of training, supervision and support. All in-depth interviews were conducted in Shona, were audio-recorded, transcribed and then translated to English. For the FGDs, these were conducted in a mixture of Shona and English and notes were taken by hand by a bilingual research assistant and where necessary translated to English. The interviews and FGDs were coded and analysed using grounded theory principles [13]. Grounded theory refers to generating theory and understandings which are ‘grounded’ or which emerge from the data that are systematically gathered and analysed, the objective being to build and expand, rather than test theory. This is appropriate as the participants’ experiences of the intervention, and perceptions of its potential benefits, were likely to incorporate a number of interacting factors that needed to be explored in this context. Using this theoretical approach, we analysed the transcripts through standard methods of thematic content analysis [14, 15]. All transcripts were initially read several times over to gain a sense of the meaning as a whole. The next step in the analysis focused on capturing the descriptive or manifest content of the articles, the obvious surface-level presentation of topic areas. Independently-identified codes were then compared, and where they addressed similar content, combined into single categories through consensus discussions. In the next step, we inductively looked for connections and relationships among the discrete codes identified in the previous step, in order to map clusters, interpret the text at greater depth, and uncover latent meanings and themes. Through a process of constant comparison, the relationships among the primary codes were integrated and condensed into the final emergent themes or categories.

### Ethical considerations

Ethical approval was granted by the Medical Research Council of Zimbabwe (MRCZ) and King’s College London Research Ethics Committee PNM/11/12-145. Written consent was gathered from all participants after explaining the purpose of the study. All digital recordings were erased following transcription, and all identifying information was removed from all transcripts. Quantitative questionnaires were kept locked after data entry was completed.

## Results

### Quantitative data

Data availability proved extremely limited, thus quantitative analyses are restricted to 2010–2014 only and to number of patients and of visits to the benches in the primary care clinics i.e. data on sessions provided in the community had not been recorded.

#### Number of patient and visits

**Fig. 1:**
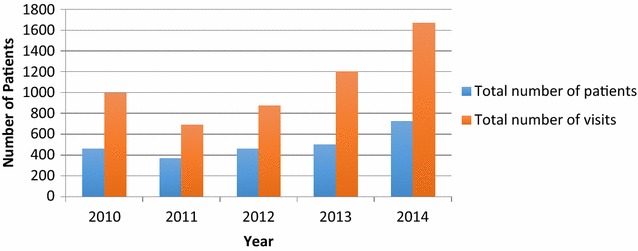
Patients seen at the Friendship Bench

Figure 1 shows the total number of patients seen at, and total visits to, the Friendship Bench. Between 2010 and 2014, 2524 patients accessed the Friendship Bench (mean per year 505 (SD 132), and the total number of visits was 5434. Following a dip between 2010 and 2011, both the number of patients and visits steadily increased from 2011 to 2014: 462 patients were seen in 2010, which increased to 728 in 2014; there were 997 total visits in 2010 growing to 1669 in 2014. Just under half of patients (48 %) attended once only. For those attending more than once, most visited twice, with only 5.7 % receiving 4 or more talking therapy sessions on the wooden benches in the clinics.

#### Proportion of visits where the LHW made onward referral for additional support (n = 656)

**Fig. 2:**
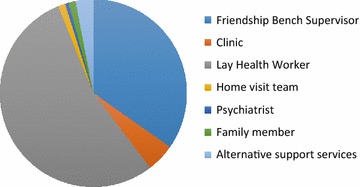
Proportion of visits where the LHWs made onward referral for additional support (n = 656)

Data on referral were available for 656/1204 (54 %) visits in 2013, (but for fewer than 40 % of total visits per year in other years thus we report on 2013 only) In 2013, although LHWs managed themselves just over half (54 %) of the 656 patient visits, they referred over one in three (228/656) to the Friendship Bench Supervisor, either just for advice or for additional assessment. QueryThey referred only four patients (0.6 % of patient visits) to the psychiatrist in 2013, compared to the 8 % observed in the 2007 pilot study [6] (Fig. 2).

#### Workforce turnover

In Mbare, all of the LHWs are female (which is the same for the whole of Harare) with primary school education only and a mean age of 62 years. In 2010, there were 15 LHWs working for the Friendship Bench. In 2014, 14 of these remained working for the program. One died in 2011.

### Qualitative data

As shown in Table 1, various themes emerged through the FGD and in-depth interviews related to the intervention’s acceptability, potential benefits, facilitating factors and potential implementation challenges.

**Table 1:** Key themes emerging from interviews and focus groups with patients, LHWs and the supervisors

Theme	Quote
Acceptability and potential benefits	
	‘Because it was like a second home you will be feeling that you have someone you can tell your problems and they listen to what I tell them.’ (Patient) ‘Because of what the granny said, I am going to go start working on my project. I had lost focus. Granny helped to open up my mind.’ (Patient) ‘…they have opened up my mind as I was a person who was in the dark…you have opened up my mind to new things that I never thought of doing.’ (Patient) ‘I was helped a lot by realising that there are some people who still see me as a human being because I was now thinking that I am not even a human being. So my mind is what I can say…is now at a better level.’ (Patient) ‘You are asked about the challenges you will be facing and you are left to express yourself freely and they also understand them help you with ideas of what to do going forth.’ (Patient) ‘I like that when I come they counsel me and tell me that I am just like a person with diabetes and that there is no need to look down upon myself. There is no need to exclude oneself from social groupings based on one’s HIV status.’ (Patient) ‘A patient seems happy by just the progress that we’ve made in that they’ll maybe repeatedly say, “Ok, I am going to try ABCD then I will come and see you on such and such a day”.’ (LHW) ‘You will leave the session feeling better because you would have talked about things.’ (Patient) ‘By talking about why they are there, I open up their mind in that I try and get them to understand that they can get help.’ (LHW) ‘Talk therapy is the best, that’s the best way, you pick up certain stuff that they are saying, you pick up the lifestyle that they are leading, you pick up certain things that maybe they used to do and they are not doing.’ (LHW) ‘Patients that suffer from *kufungisisa* (thinking too much, a Shona idiom of emotional distress)…all seem to benefit somehow.’ (LHW) ‘Through all this therapy they now manage to continue taking their medication the way they are supposed to.’ (LHW) ‘SSQ helps me to see how the person is surviving because for some they will not even be able to do their work. For some even to take their dishes outside, they cannot even do that. So it helps me to know that.’ (LHW)
Facilitating factors	
Socio-cultural appropriateness of the LHWs	‘The LHWs are respected in the community.’ (Supervisor) ‘They call us Ambuya Utano.’ (grandmother health provider) (LHW) ‘The LHW are really from the same community so they share the same social problems as their patients—like poverty, like HIV.’ (Supervisor) ‘Someone that is mature…holds a position in society, like a role model or mentor.’ (Supervisor) ‘Because through the discussions we had we get to be friends with my counsellor so much that even if I do something at home I can come to talk about it, that something happened which did not go down well with me.’ (Patient)
Patient-focused and flexible approach	‘These days, the common method of patient acquisition is patient actually going to the LHWs’ home for assistance as they are often viewed as the first port of call for health matters at all hours and time of the day.’ (Supervisor) ‘Women from our community want to sell vegetables in the morning, not sit on the Bench, so we see them in the afternoon when they are ready to talk.’ (LHW) ‘It is like planting a seed, watering it to make it grow and then meeting to remove any weeds that had sprung up.’ (LHW) ‘We hear, talk, empower the patient. We do not tell them what to do.’ (LHW) ‘She is the driver and I am the leader… [I] get in then the driver drives.’(LHW) ‘We offer counselling to empower people to help themselves… When there are changes I feel good that I have helped someone.’ (LHW)
Supportive supervision structures	‘We work very well just helping each other. If you make a mistake you are corrected and we help each other.’ (LHW) ‘We have one day aside on a Wednesday when every LHW gives feedback on a case that they’re working on, or even issues that are going on in the community.’ (LHW) ‘We work together, we work as one team.’ (LHW)
Challenges to implementation	
Socio-economic circumstances	‘A case I found difficult is when the person needs assistance immediately. One day I actually used my money after realizing that the situation was really desperate.’ (LHW) ‘They come in thinking that any problem, no matter what it is we can intervene, so we are more like a social work office sometimes. We end up being that way- where people are displaced and want to come and get assistance.’ (LHW)
Insufficient training and financial support	‘I would like to learn about such issues like dealing with a person who wants to commit suicide. …I might have my own way but I would like to be trained so that I do not do anything that lands me in jail after having let someone go ahead with committing suicide.’ (LHW) ‘There is no structure or system that’s there to support me when I’m in a situation like that…’ (Supervisor referring to lack of supervisory support for dealing with challenging clients) ‘The money that we get is not significantly different from those who do not work… There should be a difference… Apart from our uniforms, we need bags to show that we are going to work.’ (LHW)
Lack of follow and comprehensive documentation	‘The patient is the one who tells me when she wants to come back, maybe after a month.’ (LHW) ‘I do not dictate the pace.’ (LHW) ‘Promoters mentioned that they do a lot of counselling that is not documented or necessarily captured in a structured or formal context of the FB intervention.’ (Supervisor) ‘..it would be much more helpful for the patient and myself to have all those services in one place. We make patients travel from one place to the other especially for medication… maybe you can lose a patient in between.’ (Supervisor)

#### Acceptability and potential benefits

The patients, LHWs and supervisor all reported many positive views of the Friendship Bench, illustrating the intervention’s high level of personal and socio-cultural acceptability. Common sentiments expressed by the patients included the intervention assisting them to feel ‘happier’, ‘valued’, ‘less stigmatized’ and ‘less lonely’. Most described the benefits of having someone to tell their problems to and who listens, and appreciated the strong commitment of the LHWs. Several patients spoke about how the intervention helped to ‘open up my mind’. Similar kinds of opinions were expressed by the LHWs, many of whom referred to the first stage of the intervention as *kuvhura pfungwa* (Shona for ‘opening the mind’). Several elements of the intervention emerged as socially and culturally appropriate. These included the use of a screening tool which incorporated local terminology for emotional distress, especially ‘kufungisisa’ or ‘thinking too much’ which is a common idiom of emotional distress which shares features of both rumination and worry [8, 16]. Patients often spoke of the LHW as the ‘granny’ and the LHW often referred to the patients as ‘my daughter in-law’, ‘my son’, underlining the perception of the LHW as almost family members in their role in the community. Patients and staff indicated that in addition to being respected in the community, LHWs also commonly shared the same social and economic problems as their clients, and as such were seen as very suitable to deliver the intervention. A further aspect of the intervention is that it focuses on people’s problems, and on activating behaviour to get around problems. In this culture, it is very common for people with depression and CMDs to present with problems, rather than, for instance, feeling sad or anxious. Thus the link with problems adds to the intervention’s social and cultural relevance.

The benefits of the intervention were also widely highlighted by the LHWs, who frequently described the ‘many successes’ they witnessed in their patients as a result of the intervention. Health workers described how their patients frequently reported feeling ‘relieved and relaxed’ after the therapy, or that they are ‘now in a better state’ or ‘feeling better’. At the same time, delivering the intervention appears to have been of some value for the LHWs themselves, many of whom spoke about the strong sense of ‘personal reward’ and ‘satisfaction’ they feel from being agents of change. They also said that the training in mental health and PST helped them to cope better with their own lives and to be able to assist their family members. These personal benefits for the LHWs may have helped to keep them motivated, despite receiving very little remuneration for their work.

#### Facilitating factors

*Socio*-*cultural appropriateness of the lay health workers* The position and characteristics of the LHWs, who are the primary delivery agents of the intervention, emerged as a major facilitating factor for the intervention. The LHWs are deeply inserted within the same community as their patients, in most cases having lived locally for at least 15 years. Moreover, they are appointed through a selection process that takes place at community gatherings of key stakeholders, including church leaders, police, headmasters, and other community leaders. As such, they have status and possess an intimate knowledge of the local language, norms and context, and a unique social and cultural understanding of the issues facing their patients. This in turn helps them to connect with their patients, identify expressions of distress, such as *kufungisisa* (‘thinking too much’) and ‘painful heart’, and develop appropriate solutions which may be masked to an ‘outsider’. Most patients expressed trust and respect for the LHWs. The LHWs were also seen to possess certain personal attributes and competencies which further facilitated their ability to provide mental health care. They were commonly described, by patients, their supervisor and by each other as being ‘approachable’, ‘trustworthy’, ‘mature’, ‘motherly’, and possessing ‘strong listening skills’. LHWs were said to have ‘big ears, a small mouth, listening nodding whilst the person is talking’and ‘having [patients’] matters at heart’. Moreover, the LHWs connectedness to the community seemed to enhance their commitment to the intervention.

*Patient*-*focused, flexible approach* Both patients and LHWs highlighted the patient-centred and flexible nature of the intervention, whereby it could be tailored to patient needs, such as having sessions conducted in their homes or being able to self-refer. This allowed counselling to be integrated alongside the wider health promotion duties of the LHWs. Several patients expressed appreciation for being given the opportunity to be a driving force of their own treatment. Similarly, numerous LHWs described how the ‘empowering’ nature of the therapy is rewarding not only for their patients, but also for themselves, providing personal satisfaction. The LHWs emphasised that they spent a long time in the first session with the client, generating solutions as well as identifying problems ‘in case the client does not come back’.

*Supportive supervision structures* All of the LHWs reported that their peers and the higher cadres made them feel well-supported to conduct the intervention, and indicated that this contributed to the success of the intervention. This may explain the quantitative findings showing the drop in referrals they are making to the psychiatrist compared to 7 years earlier.

#### Implementation challenges

Various potential challenges emerged. For example, despite the many benefits of the LHWs’ links to the community, this could also be testing. Most reported often being approached by community members with situations that they felt required their urgent attention and personal support. Relatedly, many patients face a host of desperate problems, such as poverty, HIV/AIDS and domestic violence. Many health workers described that these situations frequently caused their patients to feel pessimistic and worthless, discouraging them from attending further sessions. Most LHWs expressed regret about limited places to refer patients, such as income-generation projects and for food handouts. Many LHWs and their supervisor indicated insufficient levels of training, such as for assessing and managing suicide and dealing with hostile patients, and the supervisor wanted supervision for herself. Moreover, although most LHWs expressed a strong commitment to the intervention, two suggested a need for further financial reward. Another potential challenge that emerged was the lack of follow-up and comprehensive documentation. Poor documentation could at least partly explain the quantitative finding of a lower than expected recorded number of follow-up appointments, although this could also be due to lack of training about need for follow-up of more depressed patients, or lack of financial incentives for LHWs. Finally, the supervisor reported the need to have a more comprehensive service in primary care, especially antidepressants drugs, to prevent loss to follow-up. She explained that primary care nurses lack training in prescribing antidepressants and drugs are often absent so the patient is asked to travel to the hospital.

## Conclusion

This case study demonstrates that a collaborative care intervention for depression and other CMDs is positively received by patients, rewarding for female community LHWs to deliver, and can be sustained over time at low cost. Having a contextually relevant cadre of health workers to deliver the psychological therapy who had status in the community and were perceived as mature and trustworthy seems to have been important, as were the supportive supervision structures, which have been identified in successful task-sharing initiatives elsewhere [17]. While our qualitative data did not reveal gender-specific issues, data has emerged from our subsequent research supporting the notion that some male clients may prefer to see a male lay community health worker of which there are none currently available in Harare [10]. PST [18, 19], combined with simple positive activity scheduling, appears an attractive option for task-shifted CMD therapy in low-resource settings as it does not require extensive training for delivery or complex skills from the therapist, and can be delivered by lay counsellors. While PST is normally a 6-session therapy, it is notable that LHWs in Harare emphasised spending a lot of time on the first session of therapy, generating solutions as well as identifying problems [10] which they said they did to make sure they offered the person something on their first meeting in order to engage them and also in case the person did not come back. This may have proved beneficial for many clients and reduced the number of subsequent appointments. Future implementation of the Friendship Bench should include ways to monitor session attendance for patients with major depression to facilitate follow-up. A user-friendly health information system would facilitate this. Having antidepressants on site in primary care, and nurses trained to prescribe for patients who do not recover after brief psychological therapy is another future need.

It is critical to establish supervision structures for any mental health care system [20]. The City of Harare Health Department has begun acting on this and provided training in mental health care to its team of District Health Promotion Officers who have started supervising LHWs in mental health work alongside supervising their usual health promotion duties. A psychologist has been providing weekly support to these District Officers for debriefing and case consultation. As part of the ongoing randomised trial of PST for depression in Harare, the research team are testing the use of a technological platform for data collection and storage, and for supervision and support of the LHWs delivering the intervention, as well as evaluating the effectiveness of the PST intervention in improving depression [7].

It is encouraging that Zimbabwe has recently launched its strategic plan for mental health services, which provides a framework for implementing and monitoring mental health programs. A key area is the human resources to be engaged both in prevention and treatment [21]. With the success of the recent Medical Education Partnership Initiative in Zimbabwe in re-building the mental health workforce {Mangezi, 2014#1159], there will be scope to work with policy makers to scale up the Friendship Bench model into other key health platforms such as HIV care and maternal care [22, 23]. Two major nongovernmental organisations are in dialogue with the Ministry of Health regarding partnership over scale-up of the Friendship Bench model to two large regions of the country.

Lessons learned from this case study would support the need for specialists to train and supervise non-specialists in brief psychological interventions for CMDs, and in managing psychiatric risk, and to train primary care nurses in antidepressant prescribing as part of any future scale up of collaborative care models. Health managers need to overcome the challenge of antidepressant drug supply in primary care, to consider incentives for task-sharing mental health work and to address ways to monitor follow-up for patients with major depression.

## Abbreviations

CMD: common mental disorders
LHWs: lay health workers
MRCZ: Medical Research Council of Zimbabwe
PHC: primary health care
PST: problem-solving therapy
SSQ: Shona Symptoms Questionnaire

## References

[1] Collins PY, Insel TR, Chockalingam A, Daar A, Maddox YT (2013). Grand challenges in global mental health: integration in research, policy, and practice. PLoS Med.

[2] Nakimuli-Mpungu E, Wamala K, Okello J, Alderman S, Odokonyero R, Mojtabai R, Mills EJ, Kanters S, Nachega JB, Musisi S (2015). Group support psychotherapy for depression treatment in people with HIV/AIDS in northern Uganda: a single-centre randomised controlled trial. Lancet HIV.

[3] Bass JK, Annan J, McIvor Murray S, Kaysen D, Griffiths S, Cetinoglu T, Wachter K, Murray LK, Bolton PA (2013). Controlled trial of psychotherapy for congolese survivors of sexual violence. N Eng J Med.

[4] Abas M, Broadhead JC, Mbape P, Khumalo-Sakatukwa G (1994). Defeating depression in the developing world: a Zimbabwean model. Br J Psychiatry.

[5] Patel V, Simunyu E, Gwanzura F, Lewis G, Mann A (1997). The Shona Symptom Questionnaire: the development of an indigenous measure of common mental disorders in Harare. Acta Psychiatr Scand.

[6] Chibanda D, Mesu P, Kajawu L, Cowan F, Araya R, Abas M (2011). Problem-solving therapy for depression and common mental disorders in Zimbabwe: piloting a task-shifting primary mental health care intervention in a population with a high prevalence of people living with HIV. BMC Public Health.

[7] Chibanda D, Bowers T, Verhey R, Rusakaniko S, Abas M, Weiss H, Araya R (2015). The Friendship Bench programme: a cluster randomised controlled trial of a brief psychological intervention for common mental disorders delivered by lay health workers in Zimbabwe. Int J Ment Health.

[8] Kidia K, Machando D, Bere T, Macpherson K, Nyamayaro P, Potter L, Makadzange T, Munjoma R, Marufu M, Araya R (2015). ‘I was thinking too much’: experiences of HIV-positive adults with common mental disorders and poor adherence to antiretroviral therapy in Zimbabwe. Trop Med Int Health.

[9] The Friendship Bench Project. [http://www.friendshipbenchzimbabwe.org/].

[10] Chibanda D, Cowan F, Machando D, Verhey R, Abas M, Lund C. Lay health workers’ experience of delivering a problem solving therapy intervention for common mental disorders among people living with HIV. Community Ment Health J. 2016. 10.1007/s10597-016-0018-2 27221123

[11] van Ginneken N, Tharyan P, Lewin S, Rao G, Meera S, Pian J, Chandrashekar S, Patel V (2013). Non-specialist health worker interventions for the care of mental, neurological and substance-abuse disorders in low- and middle-income countries. Cochrane Database Syst Rev.

[12] Chibanda D, Cowan FM, Healy JL, Abas M, Lund C (2015). Psychological interventions for common mental disorders for people living with hiv in low- and middle-income countries: systematic review. Trop Med Int Health.

[13] Strauss A, Corbin J (1998). Basics of qualitative research: selective coding techniques and procedures for developing grounded theory.

[14] Krippendorff K (1980). Content analysis: an introduction to its methodology.

[15] Graneheim UH, Lundman B (2004). Qualitative content analysis in nursing research: concepts, procedures and measures to achieve trustworthiness. Nurse Educ Today.

[16] Abas MA, Broadhead JC (1997). Depression and anxiety among women in an urban setting in Zimbabwe. Psychol Med.

[17] Honikman S, van Heyningen T, Field S, Baron E, Tomlinson M (2012). Stepped care for maternal mental health: a case study of the perinatal mental health project in South Africa. PLoS Med..

[18] Nezu AM (2004). Problem solving and behavior therapy revisited. Behav Ther.

[19] Mynors-Wallis L (1996). Problem-solving treatment: evidence for effectiveness and feasibility in primary care. Int J Psychiatry Med.

[20] Ngo VK, Rubinstein A, Ganju V, Kanellis P, Loza N, Rabadan-Diehl C, Daar AS (2013). Grand challenges: integrating mental health care into the non-communicable disease agenda. PLoS Med.

[21] Zimbabwe Ministry of Health and Child Welfare (2009). The National Health Strategy for Zimbabwe 2009–2013: equity and quality in health a people’s right.

[22] Kaaya S, Eustache E, Lapidos-Salaiz I, Musisi S, Psaros C, Wissow L (2013). Grand challenges: improving HIV treatment outcomes by integrating interventions for co-morbid mental illness. PLoS Med.

[23] Abas M, Ali G, Nakimuli-Mpungu E, Chibanda D (2014). Depression in people living with HIV in sub-Saharan Africa: time to act. Trop Med Int Health.

